# Defying Dissolution: Discovery of Deep-Sea Scleractinian Coral Reefs in the North Pacific

**DOI:** 10.1038/s41598-017-05492-w

**Published:** 2017-07-14

**Authors:** Amy R. Baco, Nicole Morgan, E. Brendan Roark, Mauricio Silva, Kathryn E. F. Shamberger, Kelci Miller

**Affiliations:** 1 Department of Earth, Ocean and Atmospheric Sciences, Florida State University, 117 N. Woodward Ave, Tallahassee, FL 32306 USA; 2 Department of Geography, Texas A&M University, College Station, TX 77843-3147 USA; 3 Department of Oceanography, Texas A&M University, College Station, TX 77843 USA

## Abstract

Deep-sea scleractinian coral reefs are protected ecologically and biologically significant areas that support global fisheries. The absence of observations of deep-sea scleractinian reefs in the Central and Northeast Pacific, combined with the shallow aragonite saturation horizon (ASH) and high carbonate dissolution rates there, fueled the hypothesis that reef formation in the North Pacific was improbable. Despite this, we report the discovery of live scleractinian reefs on six seamounts of the Northwestern Hawaiian Islands and Emperor Seamount Chain at depths of 535–732 m and aragonite saturation state (Ω_arag_) values of 0.71–1.33. Although the ASH becomes deeper moving northwest along the chains, the depth distribution of the reefs becomes shallower, suggesting the ASH is having little influence on their distribution. Higher chlorophyll moving to the northwest may partially explain the geographic distribution of the reefs. Principle Components Analysis suggests that currents are also an important factor in their distribution, but neither chlorophyll nor the available current data can explain the unexpected depth distribution. Further environmental data is needed to elucidate the reason for the distribution of these reefs. The discovery of reef-forming scleractinians in this region is of concern because a number of the sites occur on seamounts with active trawl fisheries.

## Introduction

Seamounts with deep-sea scleractinian coral reefs fall into the classification of vulnerable marine ecosystems (VMEs) and ecologically and biologically significant areas (EBSAs), thus they receive special protection status, even on the high seas^1^. Deep-sea coral reefs are vulnerable to anthropogenic stresses, including fisheries trawling, which is known to destroy reef structures^2^ with recovery likely to take decades to centuries^3, 4^. In addition, anthropogenically induced shoaling of the aragonite saturation horizon (ASH), due to global climate change and ocean acidification, is expected to lead to loss of suitable habitat for slow-growing reef-forming coral species^5^. Thus, determining the locations of deep-sea scleractinian reef sites is important in fisheries management and aids in the development of local, national and international conservation and protection policies^6^.

Deep-sea scleractinian coral reefs are found throughout the North Atlantic and the South Pacific, but thus far have not been discovered in the Central and Northeast Pacific region. Instead, dense beds of octocorals and antipatharians dominate deep-sea hard substrates in this area^7–11^. Although there are many species of scleractinians in deep waters of the North Pacific, they are predominantly solitary cup corals or individual colonies, rather than the type that accumulate into reefs^9, 12^. The general absence of observations of deep-sea scleractinian reefs in the North Pacific, despite a reasonable amount of exploration, has led to the hypothesis that reef formation in the North Pacific is “unlikely, if not impossible”^5^. This hypothesis is based on two lines of reasoning: one is the relatively shallow ASH in the North Pacific (50–600 m) compared to other regions of the worlds’ oceans; the other is that carbonate dissolution rates in the North Pacific exceed those of the North Atlantic by a factor of two^5, 13^. Consistent with these observations, habitat suitability modeling for deep-sea scleractinians also shows very little suitable habitat in the North Pacific except for some scattered locations above the ASH^14, 15^.

Despite these expectations, here we report the discovery of live scleractinian reefs at six sites in the North Pacific on seamounts of the Northwestern Hawaiian Islands (NWHI) and Emperor Seamount Chain (ESC) during an exploratory Autonomous Underwater Vehicle (AUV) survey to examine recovery of deep-sea coral communities following fisheries trawling at these sites. We compare the observed reef distribution to the available environmental data for the sites where they occur, including aragonite saturation state (Ω_arag_) and other abiotic factors, to gain insight into how reefs are able to form despite the challenging carbonate chemistry.

## Results

**Figure 1:**
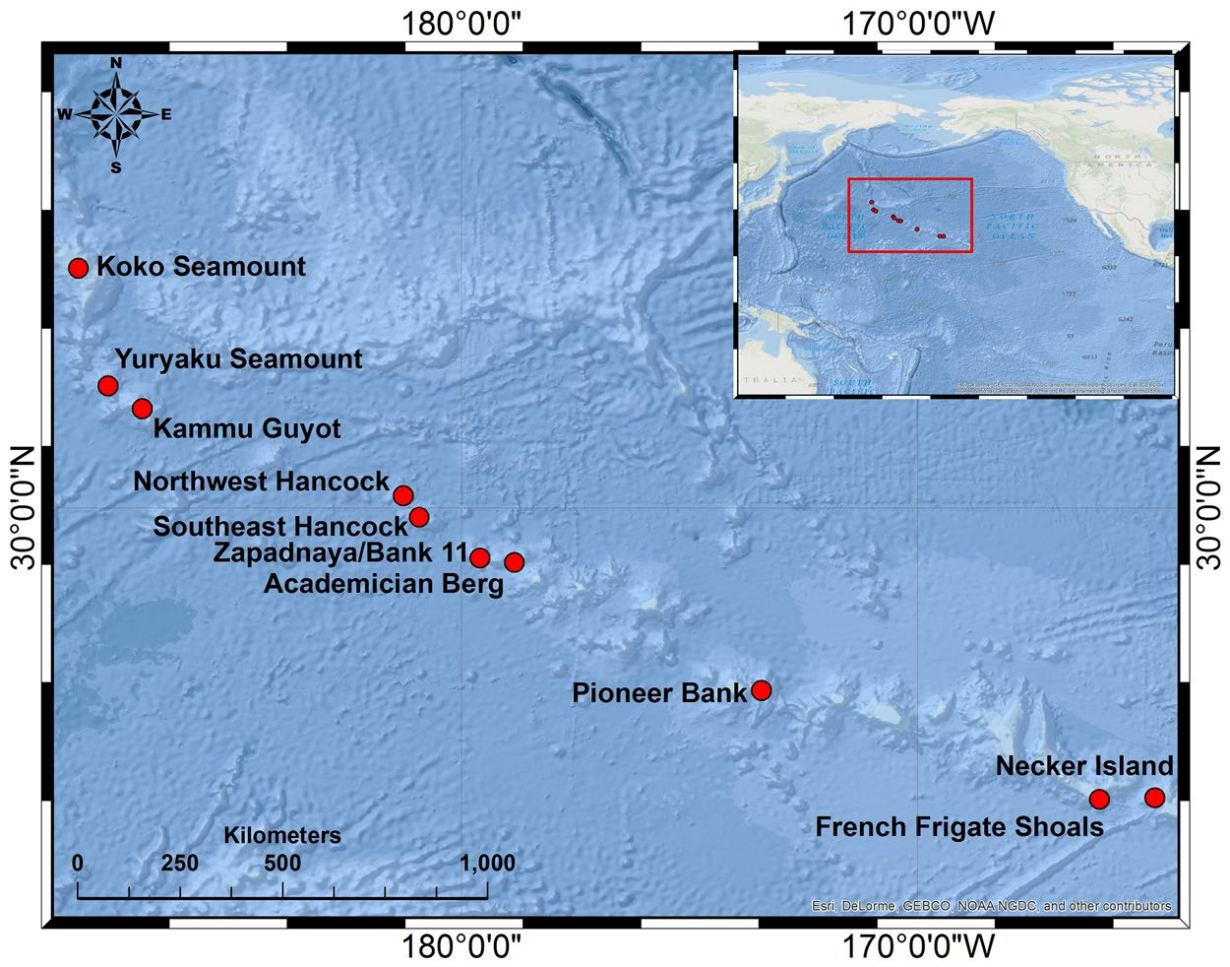
Map showing the geographic locations of surveyed sites. Figure created using ArcMap 10.4.1 with basemap provided by ESRI.

**Figure 2:**
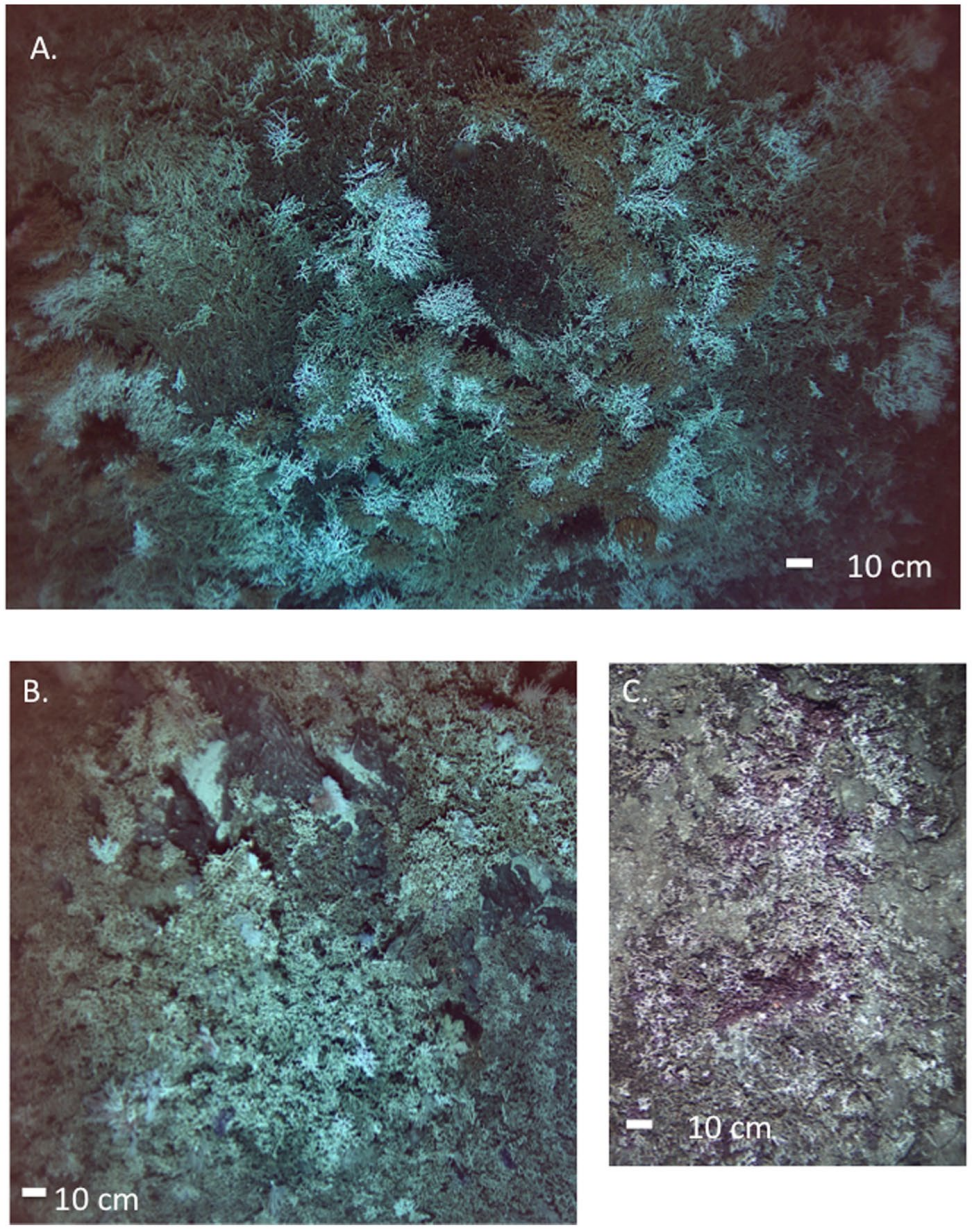
Example AUV images of observed scleractinian reefs taken ~5 m above the bottom. The reef at (**A**). 641 m on Northwest Hancock and (**B**) 637 m on Southeast Hancock, were predominantly a species with orange polyps, most likely *Solenosmilia*. (**C**) The purple scleractinian reef, most likely formed by *Enalopsammia*, at 596 m on Yuryaku. Images obtained by the authors using the AUV Sentry.

**Figure 3:**
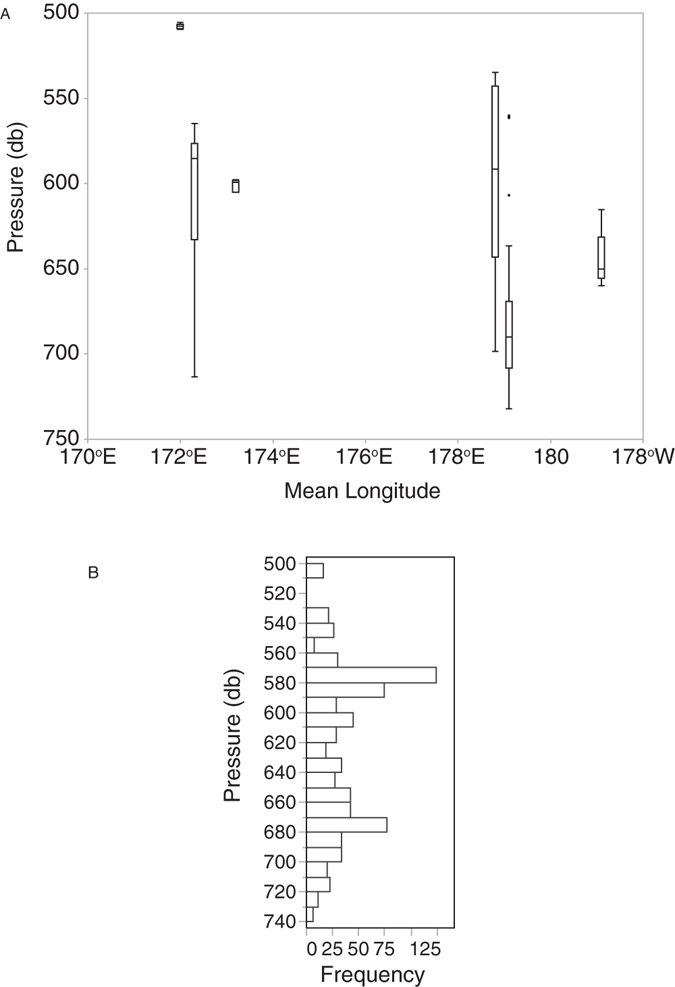
Distribution of observed scleractinian reef with depth and longitude. (**A**) Box plot based on number of images with reef present at each depth. (**B**) Histogram showing frequency of occurrence within each depth bin across sites.

Scleractinians reefs were observed on six of the 10 features within our surveyed depth range and occurred at depths of 535–732 m (the maximum depth surveyed) (Figs 1, 2 and 3, Table 1). The linear length of the reefs ranged from ~3–786 m. These values should be viewed as conservative estimates for reef length as the AUV employed in this study follows a preset course heading regardless of what is on the seafloor, as opposed to a survey with a research submersible or Remotely Operated Vehicle (ROV), which would map out the full extent of the reef.

**Table 1:** Locations surveyed as a part of this study.

Site	LATITUDE N	LONGITUDE		Scleractinian Reef Observed	CTD Cast	CTD Date	Water Depth (m)	Max CTD Depth (m)	Bottle Samples Taken
Koko Seamount	35.25	171.60	E	Yes	KM15–17 CTD-01	10/9/15	1189	1140	22
Yuryaku Seamount	32.67	172.25	E	Yes	SKQ201401S-001	11/27/14	1061	1000	24
Kammu Guyot	32.17	173.00	E	Yes					
Northwest Hancock	30.27	178.72	E	Yes	KM15–17 CTD-03	10/17/15	1216	1150	18
Southeast Hancock	29.79	179.07	E	Yes	KM15–17 CTD-04	10/20/15	1675	1500	5
Zapadnaya/Bank 11	28.90	179.60	W	No	SKQ201401S-002	12/1/14	1100	1000	20
Academician Berg	28.80	178.84	W	Yes					
Pioneer Bank	26.00	173.43	W	No	KM15–17 CTD-06	10/26/15	1508	1400	18
					SKQ201401S-008	12/5/14	1223	1210	20
French Frigate Shoals	23.61	166.01	W	No	KM15–17 CTD-07	10/31/15	1509	1000	18
Necker Island	23.65	164.80	W	No	KM15–17 CTD-08	11/5/15	1273	1000	5

All sites were surveyed at depths of 200–700 m along contours at 50 m depth intervals inclusive. Sites from Academician and southeast are part of the pre-2016 expansion boundaries of the Papahānaumokuākea Marine National Monument.

**Figure 4:**
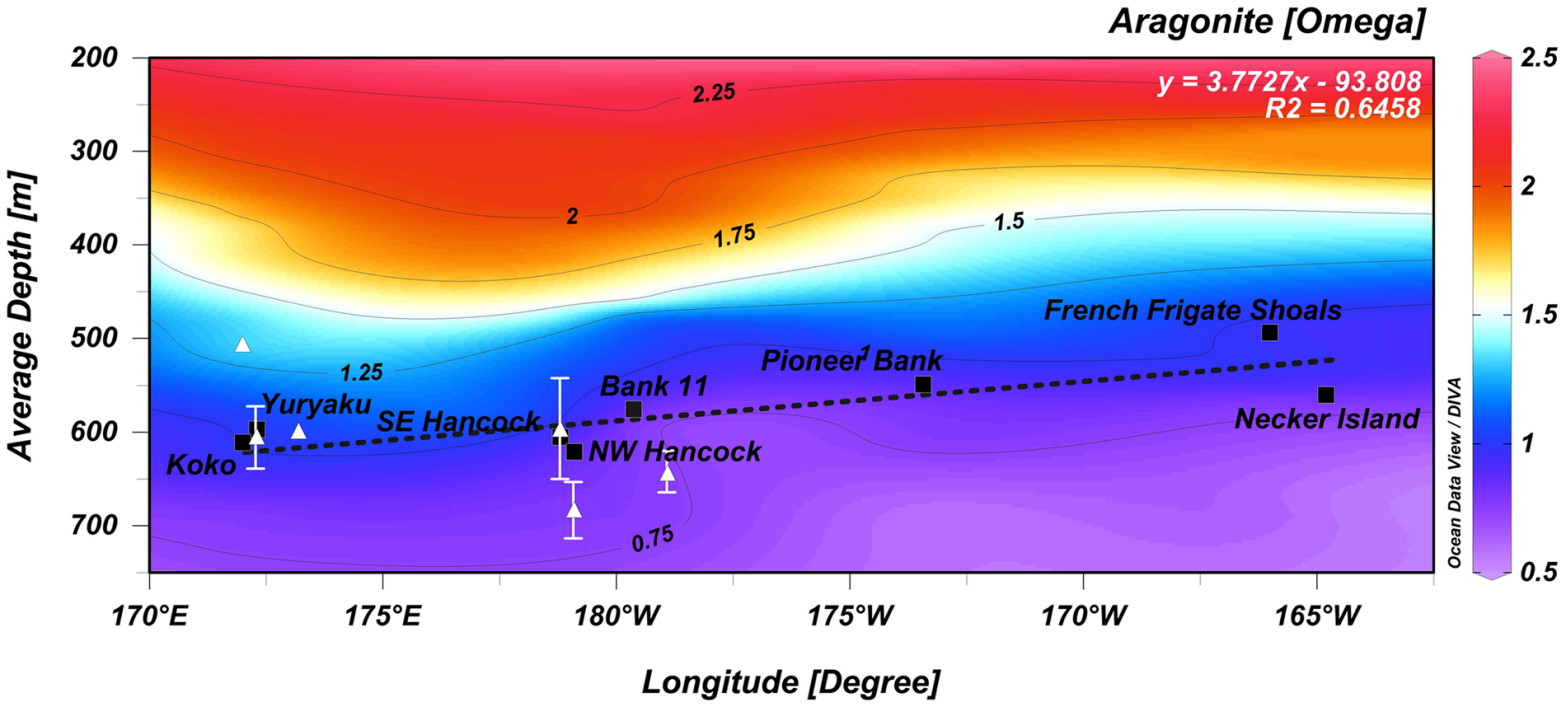
Depth of the aragonite saturation horizon (ASH) at each site with the mean depth of occurrence of scleractinian reef for each site. Black squares are mean ASH depth for each site. White triangles are mean + 1 SD for reef occurrence depth. Note that the mean depth of reefs falls below the ASH for most sites.

The range of the available environmental parameters observed at the locations at which the scleractinian reefs occurred are summarized in Table 2. The occurrence of reef was shallower moving to the northwest along the seamount chain (Fig. 3A), with a statistically significant non-parametric Spearman Rank correlation (Rho = −0.389, p < 0.0001) between depth of occurrence and longitude. The Ω_arag_ of seawater at the locations of the reef sites ranged from 0.71–1.33. The ASH deepens moving to the northwest along the NWHI and ESC chains, with the mean depth of scleractinian reefs at several sites falling below the ASH (Fig. 4).

**Table 2:** Summary of available environmental data for each seamount in this study at the sites where scleractinians were observed, including both transect and non-transect data.

	Statistic	Koko	Yuryaku	Kammu	NW Hancock	SE Hancock	Academician
**Latitude**	Mean	35.02	32.71	31.88	30.27	29.81	28.82
**Graphable Longitude**	Mean	188.01	187.76	186.85	181.28	180.93	178.92
**Pressure (db)**	Mean	507.73	608.04	600.38	598.95	684.44	645.22
	Std Err	0.38	2.15	0.75	4.40	2.81	1.62
**O**_**2**_ **(uM)**	Mean	216.64	135.97	185.53	170.49	111.14	154.14
	Std Err	0.70	1.47	0.76	2.01	1.34	0.40
**Temperature (°C)**	Mean	9.72	5.66	7.43	7.04	5.33	6.99
	Std Err	0.03	0.05	0.03	0.07	0.03	0.02
**Conductivity (siemens/m)**	Mean	3.72	3.35	3.51	3.47	3.32	3.48
	Std Err	0.00	0.00	0.00	0.01	0.00	0.00
**Chl a (μg/m** ^**3**^ **)**	Mean	0.17	0.13	0.11	0.09	0.08	0.07
	Std Err	0.00	0.00	0.00	0.00	0.00	0.00
**Backscatter (dv)**	Mean	−26.03	−22.78	−22.80	−20.86	−22.53	
	Std Err	0.12	0.16	0.50	0.47	0.20	
**Roughness**	Mean	24.07	14.71	2.65	16.56	20.21	73.25
	Std Err	0.83	0.75	0.32	0.62	0.66	0.52
**Slope (% rise)**	Mean	9.09	11.83	6.34	6.38	9.07	47.63
	Std Err	0.43	0.60	0.86	0.24	0.31	0.30
**NO**_**3**_^**−**^ **(μmol/L)**	Mean	20.95	35.59		25.32	34.79	
	Std Err	0.00	0.00		0.34	0.00	
**HPO**_**4**_^**=**^ **(μmol/L)**	Mean	1.70	2.75		2.08	2.66	
	Std Err	0.00	0.00		0.02	0.00	
**HSIO**_**3**_^**−**^ **(μmol/L)**	Mean	36.64	90.96		50.47	79.93	
	Std Err	0.00	0.00		0.94	0.00	
**NH**_**4**_^**+**^ **(μmol/L)**	Mean	0.12	0.00		0.25	0.11	
	Std Err	0.00	0.00		0.00	0.00	
**NO**_**2**_^**−**^ **(μmol/L)**	Mean	0.01	0.04		0.03	0.06	
	Std Err	0.00	0.00		0.00	0.00	
**NO**_**3**_^**−**^ **+NO**_**2**_^**−**^ **(μM)**	Mean	20.96	35.63		25.35	34.86	
	Std Err	0.00	0.00		0.34	0.00	
**TA (μmol/kg)**	Mean	2272.92	2306.85		2276.99	2297.69	
	Std Err	0.00	0.00		0.54	0.00	
**DIC (μmol/kg)**	Mean	2152.57	2272.53		2191.40	2251.53	
	Std Err	0.00	0.00		2.33	0.00	
**pH**	Mean	7.90	7.68		7.81	7.71	
	Std Err	0.00	0.00		0.00	0.00	
**pCO**_**2**_	Mean	533.50	906.50		669.01	832.00	
	Std Err	0.00	0.00		7.02	0.00	
**Ω**_**cal**_	Mean	2.09	1.12		1.67	1.22	
	Std Err	0.00	0.00		0.02	0.00	
**Ω**_**arag**_	Mean	1.33	0.71		1.06	0.78	
	Std Err	0.00	0.00		0.01	0.00

Given the altitude above the seafloor at which the images were taken, the species identification of the scleractinians in most images could not be determined. The depth distribution curve for observed reef structure across sites (Fig. 3B) was bimodal suggesting at least 2 species are present. Based on the purple coloration of the polyps and colony morphology, *Enalopsammia rostrata*, a species known to occur at other sites in the Hawaiian Archipelago as individual colonies, was the likely reef-former at Academician, Kammu, Northwest Hancock and Yuryaku Seamounts (Fig. 2C). A second morphotype with orange polyps that is likely *Solenosmilia variabilis* was the abundant species on Koko and both Hancock Seamounts (Fig. 2A,B). At several sites both morphotypes occurred in the same images. We could not do a quantitative analysis of the distribution of either species separately because in many images the resolution was not sufficient to distinguish between them.

**Figure 5:**
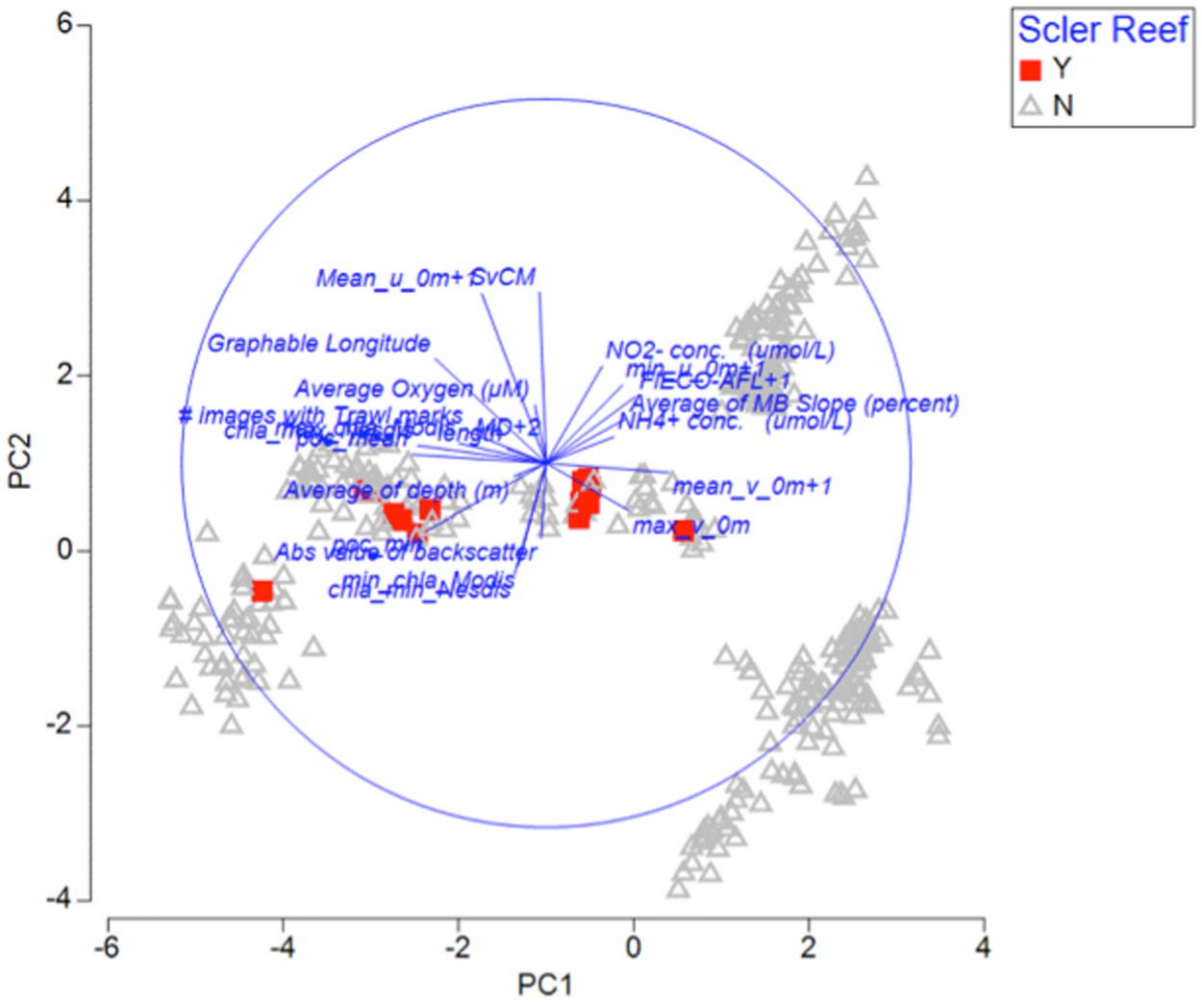
PCA plot of available environmental data after removal of variables that were more than 90% correlated. Each point represents a single 1 km AUV transect.

**Figure 6:**
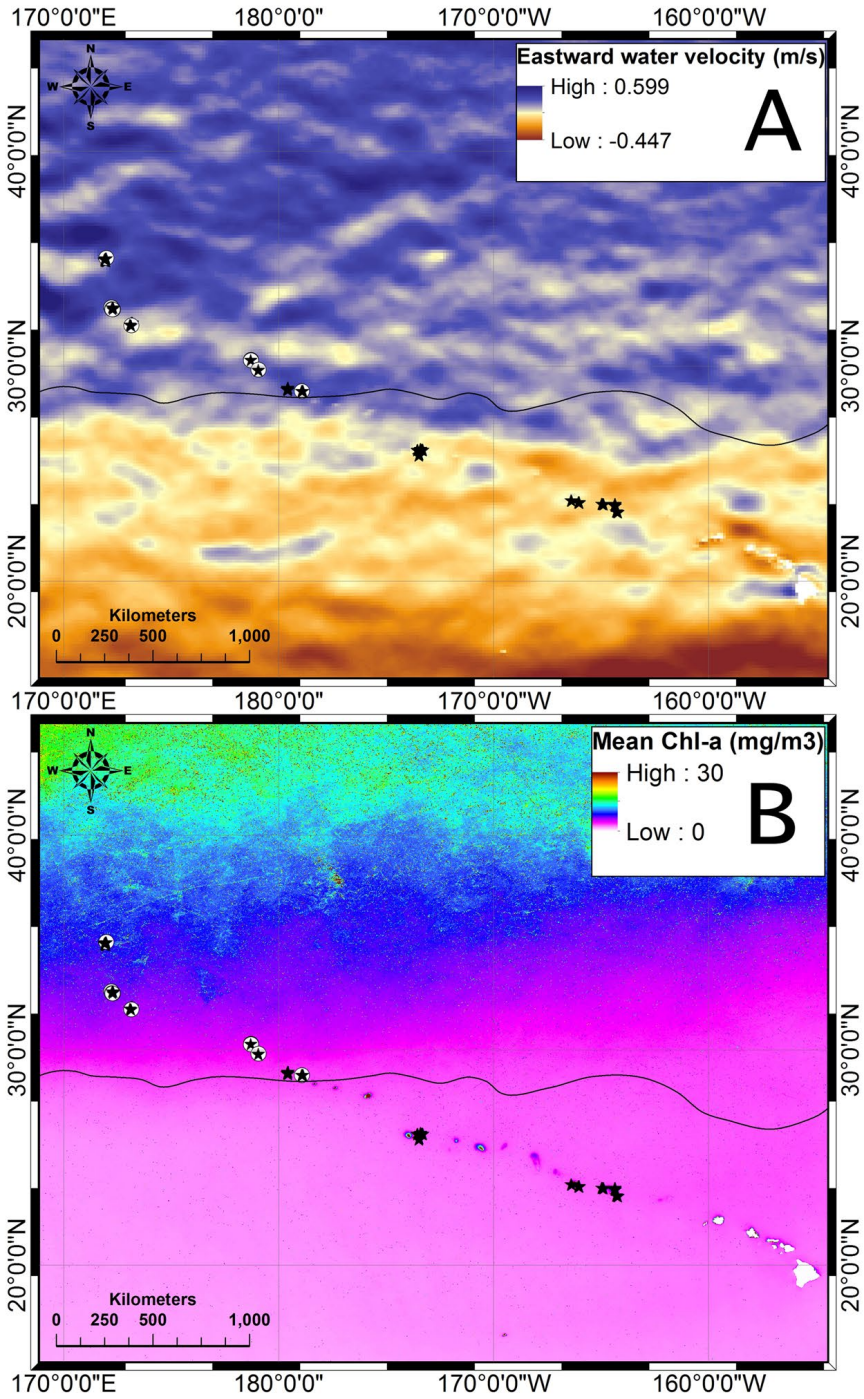
(**A**) One year average (2015–2016) raster of the eastward surface water velocity (u) across the study area. Positive values (blue) show predominance of eastward currents. Negative values (red) represent westward prevailing currents. Figure created using ArcMap 10.4.1 using HYCOM data as described in the methods. (**B**) Eight year average composite raster of surface chlorophyll-a concentration (2008–2016). Black contour delimits 0.07 mg/m^3^ chl-a concentration. Figure created using ArcMap 10.4.1 using satellite data obtained from Aqua-MODIS satellite available on the ERDDAP^42^ data set as described in the methods. In both panels white circles indicate the location of transects with scleractinian reefs and stars indicate transects which did not include reef structures.

Principle Components Analysis (PCA) was performed to determine which environmental factors correlate most strongly with the distribution of scleractinian reefs. PCA shows that each site groups strongly with other points from the same seamount (Fig. 5). Scleractinian reefs sites occupy a small range of PCA axis 2, but a broad range of each of the other four PCA axes. PCA axis 2 was most strongly correlated with sound velocity and the east-west component of the surface current velocity (‘u’), suggesting currents may be the most influential of the measured factors on the occurrence of scleractinian reefs (Table 3). There is a clear shift in current direction near the location where the reefs begin, with westward currents dominating to the southeast and eastward currents dominating to the northwest (Fig. 6A).

**Table 3:** Summary of PCA analyses of all transects.

Eigenvectors					
(Coefficients in the linear combinations of variables making up PC’s)					
Variable	PC1	PC2	PC3	PC4	PC5
Graphable Longitude	−0.304	0.287	−0.134	0.043	−0.053
Average of Depth (m)	−0.089	−0.036	−0.114	**0.594**	**0.316**
Average Oxygen (μM)	−0.031	0.16	−0.016	**−0.565**	**−0.386**
Mean Direction	−0.001	0.054	−0.07	−0.069	−0.094
No. images with Trawl marks	−0.217	0.088	0.102	0.167	−0.331
Transect Length	−0.108	0.039	−0.037	0.098	−0.088
Average of MB Slope (%)	0.218	0.122	−0.187	0.027	−0.027
Backscatter	−0.014	−0.206	−0.117	−0.235	**0.306**
Minimum POC	−0.323	−0.183	0.264	0.033	−0.028
Mean POC	**−0.367**	0.023	−0.048	−0.06	0.061
Max Chl from Modis	−0.241	0.055	0.075	0.123	−0.113
Min Chl from Modis	−0.077	−0.282	0.293	−0.041	−0.232
Min Chl from Nesdis	−0.088	−0.306	**0.432**	−0.054	0.027
Max Chl from Nesdis	**−0.352**	0.048	0.15	−0.064	0.123
Mean v at 0 m	**0.336**	−0.026	0.21	0.154	−0.163
Max v at 0 m	0.228	−0.13	−0.267	0.087	−0.31
Min u at 0 m	0.209	0.214	−0.005	−0.218	**0.314**
Mean u at 0 m	−0.177	**0.466**	−0.076	−0.017	0.083
Sound velocity (m/s)	−0.018	**0.47**	0.246	0.103	−0.067
Fluroescence (mg/m^3^)	0.244	0.181	0.239	0.31	−0.277
NH_4_^+^ (μmol/L)	0.186	0.072	0.361	−0.09	**0.336**
NO_2_^−^ (μmol/L)	0.154	0.265	**0.402**	−0.075	0.136
Eigenvalues					
PC	Eigenvalues	%Variation	Cum.% Variation		
1	6.8	31	31		
2	3.2	14.6	45.5		
3	2.19	10	55.5		
4	1.95	8.9	64.4		
5	1.56	7.1	71.5		

Bold indicates the highest absolute values within each PC axis. The number of variables analyzed was reduced by removing one variable from each pair with greater than a 90% correlation. Total Alkalinity was also removed to allow more seamounts to be included in the analyses. (It was not strongly correlated to any PCA axis in preliminary data analyses).

## Discussion

Extensive explorations from Bank 11 to the southeast in the NWHI had so far not discovered any deep-sea coral reefs, even though both submersible dives and ROV explorations had included the same depth zones at several different islands and seamounts^11^. Indeed, it was thought to be improbable that scleractinian reefs would occur in the North Pacific due to carbonate chemistry that is expected to make reef formation and accumulation challenging. The carbonate dissolution rate in the North Pacific peaks between 400–600 m then decreases rapidly with depth^13^. This depth range overlaps with the relatively shallow range of the ASH in the North Pacific (50–600 m). Thus, theoretically, the most challenging depth range for reef formation in the North Pacific would begin somewhere around 600 m depth, and continue deeper. Yet, here we document observations of reefs at six seamounts in the NWHI and ESC at depths of 535–732 m.

This begs the question, how is it that the reefs can occur at these sites? One potential insight comes from a closer examination of the ASH depth in Feely *et al*.^13^, which indicates that the ASH becomes deeper moving to the northwest along the NWHI (confirmed by our results in Fig. 4). This suggests that, if the reef-forming species have a narrow depth range tolerance, the likelihood of finding reefs would increase moving to the northwest along the NWHI. That is indeed what we found, reefs were observed on every feature explored to the northwest of Academician Berg except Bank 11. However, if the ASH were the main factor governing the distribution of these corals, we would expect their depth range to stay the same or deepen moving northwest along the chain, paralleling the ASH. Instead the observed depth range becomes shallower. Further, a number of reef sites also occur below the ASH, with measured Ω_arag_ values of 0.71–1.33, together these suggest the ASH is not the primary controlling factor for the distribution of reefs.

Although the ASH would be expected to be a limiting factor for calcifying deep-sea coral species that produce aragonite^5^, results are mixed with respect to the response of calcifying species to low Ω_arag_ and pH. Supporting the idea that the ASH would be limiting, laboratory CO_2_ exposure experiments have shown that calcification rates of deep-sea corals decrease with decreasing Ω_arag_^16–20^, that deep-sea corals produce skeletons that are more susceptible to erosion under low Ω_arag_ conditions^21^, and that net dissolution occurs in live corals in undersaturated (Ω_arag_ < 1) waters^18–20^. However, other experimental studies have shown no response in calcification and respiration rates to changing Ω_arag_^19, 22, 23^, and it has been well documented that deep-sea corals can live and calcify in undersaturated waters^24, 25^. For example, Thresher *et al*.^24^ also found a number of deep-sea corals on seamounts south of Tasmania living below the ASH and calcite saturation horizon. An important exception that they note though is that the reef-forming scleractinians, including *Enalopsammia* and *Solenosmilia*, were limited to depths “saturated or near saturated” with respect to aragonite. In contrast, we find scleractinian reefs in waters with Ω_arag_ well below 1 in the NWHI and ESC.

In fact, a number of studies have suggested that some deep-sea coral species have physiological mechanisms to compensate for undersaturation and to maintain their calcifying processes and internal pH^25–27^. For example, Thresher *et al*.^24^ postulated that the non-reef forming scleractinian corals below the ASH might be able to survive due to high regional productivity resulting in an abundant food supply. This food supply could provide the excess energy needed for calcification in undersaturated waters. In contrast, Maier *et al*.^18^ found that feeding in laboratory experiments did increase calcification rates of the Mediterranean deep-sea coral *Madrepora oculata* at ambient Ω_arag_, but feeding had no effect on calcification rates under low Ω_arag_ conditions. While the authors attribute this to the small fraction (1–3%) of the total metabolic energy demand required for calcification in *Madrepora oculata*, this does not explain why feeding appears to have enhanced calcification at ambient Ω_arag_. Georgian *et al*.^28^ found that net calcification, respiration and prey capture rates of *Lophelia pertusa* from the Gulf of Mexico decreased with decreasing pH and Ω_arag_ but, in the same species from Norway, respiration and prey capture rates increased, and calcification only decreased slightly with declining Ω_arag_. These results suggest that local environmental conditions, including food supply, could result in regional differences in the ability of deep-sea corals to adapt and/or acclimate to ocean acidification.

While the main Hawaiian Islands and much of the NWHI are located in oligotrophic waters, there is a transition to higher chlorophyll waters moving to the northwest, characterized by a front referred to as the Transition Zone Chlorophyll Front (TZCF). The position of this front varies seasonally and annually, crossing the Archipelago somewhere near 180° longitude in the years it reaches its maximum extent^29^, very close to our southeastern most site with reefs, Academician Berg at 178.84°W. Figure 6B shows the annual mean surface water chlorophyll concentration across this region averaged over the period 2008–2016. Sites with reefs have higher mean annual chlorophyll than those without. Thus higher chlorophyll, while not an explanation for why the corals occur shallower, could at least be a plausible explanation for why these reefs occur from Academician and further northwest. Consistent with this, PCA analyses indicates that particulate organic carbon (POC) and Chlorophyll a (Chl-a) were most strongly correlated with PCA axis 1 (Fig. 5, Table 3). However, sites with scleractinians were distributed broadly across the PCA 1 axis, suggesting that potential food supply alone cannot explain the distribution of these reefs.

Of the five PCA axes, transects with scleractinian reefs occupied a very narrow range of PCA axis 2 when graphed relative to the four other axes. PCA axis 2 was most strongly correlated with sound velocity and the east-west component of surface currents. This suggests there is a very narrow range of current velocity needed for the survival of the deep-sea scleractinian reef-forming species. That currents might be tied to the distribution of deep-sea coral reefs is not at all surprising because the occurrence of corals near topographic highs with maximum current velocities has been recognized since some of the earliest work on seamounts^30^. However many other transects without reefs also fell within the same range of the PCA 2 axis as the scleractinian reef sites, suggesting currents are also not the only factor critical for reef occurrence. In addition, surface current data may not necessarily represent what the corals are experiencing at depth and alone could not explain why the depth distribution of reefs decreases to the northwest.

More research is clearly needed to explain the distribution of these reefs. Ideally we would use species distribution modeling to analyze the factors most correlated with the distribution of these species as well as to determine the locations of possible areas of suitable habitat^14, 15, 31^, but we currently do not have high enough resolution data for key parameters that would go into such modeling, in particular backscatter, *in situ* currents, and other important factors.

The occurrence of the observed coral reefs in the NWHI and ESC was limited to sites located outside the pre-2016 expansion boundaries of the Papahānaumokuākea Marine National Monument, including several seamounts with active trawling. This raises concern for protection of these fragile habitats and the question of what their extent might have been in this region prior to trawling. Additionally, the long-term response to increasing seawater CO_2_ levels by marine calcifying organisms susceptible to ocean acidification^32^ is a major concern for the management and conservation of corals. Determining the tie of these species distributions to Ω_arag_ is becoming time-critical because the ASH is expected to continue shoaling in this region as anthropogenic CO_2_ continues to increase in the atmosphere and ocean^5, 33^, which could further limit suitable habitat and thus threaten reef-forming scleractinians.

## Methods

As part of a project examining the recovery potential of deep-sea coral communities impacted by trawling in the NWHI and ESC, we explored 10 seamounts at depths of 200–700 m using the AUV *Sentry* during cruises in 2014 and 2015 (Table 1, Fig. 1). On each seamount three sides of the seamount were surveyed and on each side, replicate photo transects of 1000 m length were conducted. During image transects, the speed of the AUV was 0.5–0.7 m/s and the vehicle was approximately 5 m above the bottom.

All images taken during and between transects were scanned for presence of scleractinian reefs using enlarged thumbnails on a Macintosh computer. Images with scleractinians were then viewed more closely in Preview v 7.0 (Apple Inc.) and categorized into one of four categories – “Definite live reef” – which included images of reef that had visible open polyps; “Likely live reef” – images of reef with no visible polyps but areas of lighter colored skeleton similar to the skeleton found on the colonies with live polyps. Generally these images also occurred in proximity to images with definitely live reef. “Reef patches” – areas with smaller clusters of colonies that appeared live but did not form a continuous reef structure, and “Coral rubble” – areas with an accumulation of scleractinian coral skeleton fragments but no evidence of live colonies. For the environmental analyses, only images that fell into the first three categories were used.

Environmental data for the seamounts were obtained from several sources. A Seabird SBE49 Conductivity-Temperature-Depth (CTD) with a Seapoint optical backscatter (OBS) sensor and a Anderaa optode (model 4330) oxygen concentration sensor on the AUV *Sentry* provided *in situ* temperature, salinity, depth, dissolved oxygen and turbidity data that was linked directly to each image that was taken.

A Sea-Bird Electronics, Inc. (SBE) 911*plus* conductivity-temperature-depth (CTD) instrument with a rosette of twenty-four 10 L Niskin bottles was used to record water column environmental profiles and collect water samples. The CTD included sensors to measure temperature, salinity, pressure, sound velocity (Chen-Millero [m/s]), dissolved oxygen (SBE 43 [μmol/l]), and fluorescence (Wetlab ECO-AFL/FL [mg/m^3^]). The hydrocasts were conducted down to 800 to 1200 m water depth as close to the AUV survey areas as possible, typically within 1–4 km. At each site, at least one “high resolution” profile was completed with water samples taken at uniform standard depths (5, 25, 50, 75, 100, 125, 150, 200, 250, 300, 350, 400, 500, 600, 700, 800, 900, 1000, 1200 m) with the final sample taken as near to the bottom as possible (~25 m off of the bottom). At least 3 Niskin bottles were used as duplicate samples. Additional “low resolution” profiles with water samples taken at 5, 125, 300, 700 and 1000 m and CTD only characterizing profiles were made at additional locations around the site to characterize the local heterogeneity. All hydrographic data were processed using the SBE data processing software using current manufacture calibrations. The locations, water depths, and sampling depths are listed in Table 1.

Dissolved nutrient seawater samples collected at each CTD station were stored in acid-cleaned high-density polyethylene 20 mL scintillation vials. Vials were rinsed and triple filled with sample seawater before saving the final sample for analysis. Samples were immediately frozen until analyzed at the Geochemical and Environmental Research Group at Texas A&M University, College Station. Nutrient samples were analyzed on an Astoria-Pacific auto-analyzer using nitrate/nitrite/silicate methods based on Armstrong *et al*.^34^; phosphate methods based on Bernhardt and Wilhelms^35^; and ammonium methods based on Harwood and Kuhn^36^. The dissolved inorganic nitrogen (DIN) concentrations were calculated as the sum of nitrite, nitrate, and ammonium concentrations. Analytical detection limits were 0.01 μM for phosphate, 0.003 μM for nitrite, 0.05 μM for nitrate and silicate, and 0.08 μM for ammonium.

Discrete water samples were collected from Niskin bottles for total alkalinity (TA) and dissolved inorganic carbon (DIC) measurements into 250 ml borosilicate glass bottles by rinsing and triple filling, taking care to prevent bubbles in the sampling tubing and bottles. Water samples were preserved with 100 µl of saturated mercuric chloride (HgCl_2_) and ground glass stoppers were sealed with Apeizon grease and electrical tape. TA and DIC analyses were performed with a Versatile Instrument for the Determination of Titration Alkalinity (VINDTA) produced by Marianda Marine Analytics and Data. The VINDTA uses coulometric titration for DIC analysis and an open cell potentiometric titration for TA analysis. DIC and TA measurements were standardized with certified reference materials obtained from Andrew Dickson at Scripps Institution of Oceanography^37, 38^. Analyses of replicate samples yielded a mean precision of approximately 2.5 μmol kg^−1^ and 2 μmol kg^−1^ for DIC and TA analyses, respectively. The full seawater CO_2_ system was calculated using *in-situ* salinity, temperature, TA, and DIC data using an Excel Workbook Visual Basic for Applications translation of the original CO2SYS program^39^ by Pelletier, Lewis, and Wallace at the Washington State Department of Ecology, Olympia, WA. The CO2SYS program was run with carbonate constants from Mehrbach *et al*.^40^ refit by Dickson and Millero^41^.

Surface Chl-a and POC were extracted from the National Oceanic and Atmospheric Administration’s Environmental Research Division’s Data Access Program (ERDDAP) Data Set^42^. Surface current (zonal (u, east-west) and meridional (v, north-south)) data were extracted from HYCOM (HYbrid Coordinate Ocean Model). Daily values of surface current data (1/12 degree resolution) were extracted from January 1, 2015 to January 1, 2016. Monthly composites of surface Chl-a (0.025 degree resolution) and POC (4 km resolution) data were derived from Aqua-MODIS satellite from January 2008 to December 2016. All data (*.NetCDF) were imported into Matlab for quality control and then exported into ArcMap (10.4.1)^43^ to calculate raster statistics (average, maximum, and minimum) for all data sets.

Seafloor bathymetric and backscatter data was collected by Kongsberg EM122 multibeam echo sounders during cruises in 2014–2015 on R/V *Falkor*, R/V *Sikuliaq*, and R/V *Kilo Moana*. Processed bathymetric data were imported into QGIS and then analyzed for slope and roughness. Slope was calculated as a percentage and measures the inclination to the horizontal. Roughness is calculated by the largest bathymetric difference between a central pixel and its surround cells in a 3 × 3 grid^44^. Point vector layers of AUV *Sentry* transects were overlaid onto the slope, roughness, and backscatter layers and data from those layers were extracted into the transect points. Values were then averaged for a final per transect measurement.

Summary statistics for the environmental data and determination of depth distributions were completed in JMP pro v 12.0.1^45^. These analyses included all images with scleractinian reefs, regardless of whether they were taken on a transect or during transits between transects. Oceanographic CTD profiles were plotted using Ocean Data View software^46^.

To try to tease out what environmental factors were most correlated with scleractinian distributions, we performed a PCA including all transects from the AUV *Sentry* regardless of scleractinian presence or absence. We did not include data for transits between the transects in the PCA. Environmental data for the PCA plot were log (x + 1) transformed and normalized in Primer v. 7.0^47^. Draftsmen plots showed a strong correlation among many of the environmental variables even after transformation and normalization. Thus the environmental data was reduced by eliminating 1 environmental variable out of each pair with a >90% correlation (Supplemental Table 1). Correlational PCA was then completed in PRIMER using 22 environmental variables.

## Electronic supplementary material

Supplemental Table 1.
